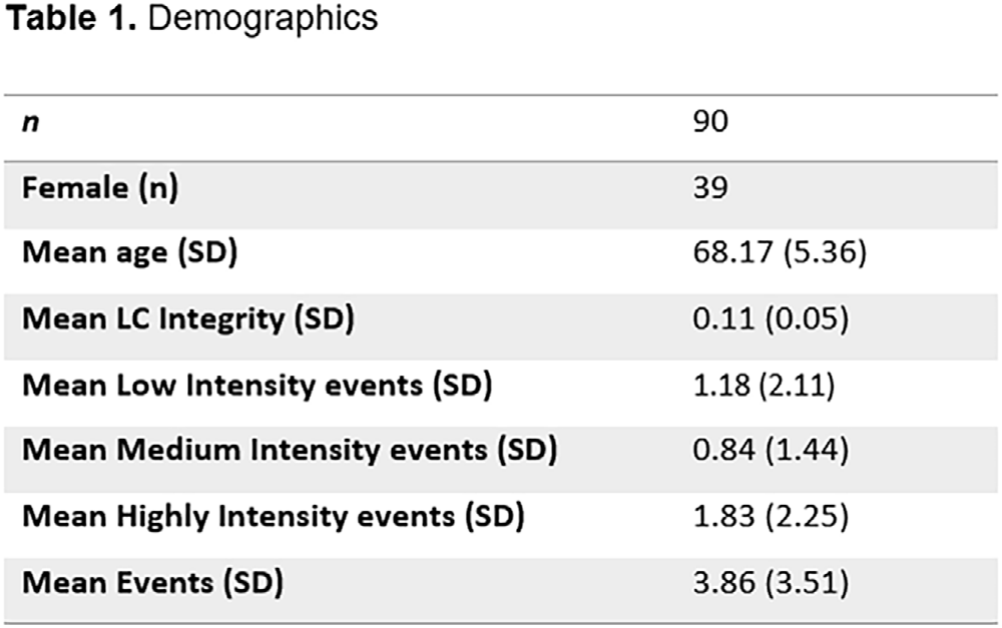# Assessing the impact of trauma on Locus Coeruleus integrity in healthy older adults ‐ Preliminary results

**DOI:** 10.1002/alz.094929

**Published:** 2025-01-09

**Authors:** Marina Leiman, Guruprasath Gurusamy, Max Dünnwald, Niklas Behrenbruch, Beate Schumann‐Werner, Eóin N. Molloy, Svenja Schwarck, Berta Garcia‐Garcia, Anne Hochkeppler, Larissa Fischer, Kathrin Baldauf, Peter Schulze, Enise I Incesoy, Michael C. Kreissl, Anne Maass, Matthew Betts, Emrah Düzel, Dorothea Hämmerer

**Affiliations:** ^1^ Institute of Cognitive Neurology and Dementia Research (IKND), Otto‐von‐Guericke University, Magdeburg Germany; ^2^ German Center for Neurodegenerative Diseases (DZNE), Magdeburg Germany; ^3^ Otto‐von‐Guericke University, Magdeburg Germany; ^4^ Division of Nuclear Medicine, Department of Radiology & Nuclear Medicine, Faculty of Medicine, Otto von Guericke University, Magdeburg Germany; ^5^ German Center for Neurodegenerative Diseases (DZNE), Berlin Germany; ^6^ Center for Behavioral Brain Sciences (CBBS), Magdeburg Germany; ^7^ Institute of Cognitive Neuroscience, University College London (UCL), London United Kingdom; ^8^ University of Innsbruck, Innsbruck Austria; ^9^ Wellcome Centre for Human Neuroimaging, University College London (UCL), Queen Square Institute of Neurology, London United Kingdom

## Abstract

**Background:**

Previous studies have examined the impact of post‐traumatic stress disorder and chronic stress on the Locus Coeruleus‐Noradrenergic System (LC‐NA) revealing significant neurobiological alterations (Aston‐Jones & Cohen, 2005; McCall et al., 2015). However, while animal studies have yielded valuable insights regarding the effects of traumatic experiences on the LC‐NA system, translation to human models remains relatively underexplored. Thus, our study aims to address this gap by investigating the relationship between traumatic experiences and LC‐NA integrity in human participants. Here, we present preliminary results on the association between traumatic events, resilience, and LC integrity.

**Method:**

We recruited 90 healthy older adults (mean age: 68.17 ± 5.36) from the SFB‐1436 Cohort. We acquired neuromelanin‐sensitive 3T MRI scans using a Magnetization Transfer Contrast (MTC) sequence optimized for enhancing the visibility of the LC. We performed LC segmentation using an automated approach (based on Dünnwald et al., 2021) and assessed traumatic experiences using a self‐reported modified version of the Life Events Checklist for DSM‐5 (LEC‐5; Weathers et al., 2013). We conducted a linear regression analysis to examine whether traumatic experiences could predict LC integrity.

**Result:**

A linear regression analysis was carried out to assess if traumatic events, age, and sex could predict LC integrity in healthy older adults. The results suggest a trend towards significance for individuals reporting Low traumatic events (*p* = 0.059) and Highly traumatic events (*p* = 0.082), indicating potential positive relationships with LC integrity, while controlling for age and sex. The overall model explained a small, non‐significant proportion of the variance in LC integrity (Adjusted R‐squared = 0.028, *p* = 0.1927).

**Conclusion:**

The analysis indicates a potential association between LC integrity and traumatic events, particularly for low and high levels of trauma. However, the overall model including traumatic events, age, and sex did not explain a significant proportion of interindividual differences in LC integrity. This suggests that while there may be trends indicating associations, they are not robust enough to be considered statistically significant in this sample. Further mediational and moderational analyses will be conducted to gain a deeper understanding of interindividual differences in this regard.